# Effects of Adaptation Rate and Noise Suppression on the Intelligibility of Compressed-Envelope Based Speech

**DOI:** 10.1371/journal.pone.0133519

**Published:** 2015-07-21

**Authors:** Ying-Hui Lai, Yu Tsao, Fei Chen

**Affiliations:** 1 Research Center for Information Technology Innovation, Academia Sinica, Taipei, Taiwan; 2 Department of Electrical & Electronic Engineering, South University of Science and Technology of China, Shenzhen, China; MRC Institute of Hearing Research, UNITED KINGDOM

## Abstract

Temporal envelope is the primary acoustic cue used in most cochlear implant (CI) speech processors to elicit speech perception for patients fitted with CI devices. Envelope compression narrows down envelope dynamic range and accordingly degrades speech understanding abilities of CI users, especially under challenging listening conditions (e.g., in noise). A new adaptive envelope compression (AEC) strategy was proposed recently, which in contrast to the traditional static envelope compression, is effective at enhancing the modulation depth of envelope waveform by making best use of its dynamic range and thus improving the intelligibility of envelope-based speech. The present study further explored the effect of adaptation rate in envelope compression on the intelligibility of compressed-envelope based speech. Moreover, since noise reduction is another essential unit in modern CI systems, the compatibility of AEC and noise reduction was also investigated. In this study, listening experiments were carried out by presenting vocoded sentences to normal hearing listeners for recognition. Experimental results demonstrated that the adaptation rate in envelope compression had a notable effect on the speech intelligibility performance of the AEC strategy. By specifying a suitable adaptation rate, speech intelligibility could be enhanced significantly in noise compared to when using static envelope compression. Moreover, results confirmed that the AEC strategy was suitable for combining with noise reduction to improve the intelligibility of envelope-based speech in noise.

## Introduction

A cochlear implant (CI) is currently the only electronic device that can provide a sense of sound to people with profound-to-severe sensorineural hearing loss (SNHL). According to an investigation by the US Food and Drug Administration published in December 2012, approximately 324,200 people worldwide have received CIs [1], and this number will continue to increase in the foreseeable future. Although CI devices can considerably improve the hearing abilities of individuals with profound-to-severe SNHL in a quiet environment, there still exists a large gap between the efficacies of a CI in quiet and in noise and/or reverberation [2, 3]. In a CI device, acoustic signals are received by a microphone and fed into a speech processor, which captures multichannel temporal envelopes of the input signals and then electrically stimulates the residual auditory nerves of CI users [4]. The dynamic range (DR) of the acoustic signals in normal conversational speech is typically 30–50 dB [5, 6]; however, the DR of electrical stimulation is limited to 5 dB for CIs due to biological constraints [7]. The large discrepancy between these DR values necessitates the use of a compression scheme.

Several DR compression strategies have been proposed, with the most common implemented in the present CI devices being static envelope compression (SEC). The SEC strategy uses a fixed compression function [i.e., compression ratio (CR)] to perform compression [8], and a logarithmic function is usually used in this transformation because it matches the amplitudes between the acoustic loudness and the electrical signal [9, 10]. Previous studies have indicated that the DR of temporal envelope is an important factor determining the speech intelligibility for CI users [6, 11, 12], and this will be affected by the CR. More specifically, the use of a larger CR in SEC will reduce the DR of temporal envelope. Zeng et al. [6] and van Hoesel et al. [11] found that a temporal envelope with a small DR resulted in poor speech recognition performance in both quiet and noisy conditions, whereas a large DR could facilitate the speech recognition of CI users. While this fixed compression function confines the overall electrical current applied to the CI within a small DR, this strategy is not optimized in terms of making best use of the small hearing DR for speech perception.

Several adaptive strategies have been proposed to address the above challenge. One notable example is the advanced combinational encoder (ACE) sound coding strategy [13, 14]. The ACE strategy adopts the front-end automatic gain control (AGC) and the adaptive dynamic-range optimization (ADRO) [15–17] scheme to adjust the gain and section of the signals in the suitable DR for each frequency band, ensuring that speech components are always presented at a comfortable listening level. Next, a static loudness growth function (LGF) is applied to enable the CI recipient’s loudness perception to match that of normal hearing (NH) individual for changes in sound intensity [13, 14]. In this way, the selected section of input DR can be presented at a comfortable level for CI recipients. James et al. [15] investigated the effect of ADRO on speech perception among CI subjects, and their results indicated that this strategy provided significant benefits over traditional SEC when speech was present in a quiet environment, but no significant benefit in noisy conditions. Dawson et al. [16] found that ADRO could provide benefits to children in both quiet and noise. In addition, Blamey [18] evaluated the performance of ADRO in bimodal CI users [i.e., using a hearing aid (HA) in one ear and a CI in the other], and their results showed that ADRO processing was particularly well suited to bimodal and hybrid stimulations that combined electrical and acoustic stimulations in opposite ears or in the same ear.

More recently, Lai et al. proposed a new adaptive envelope compression (AEC) strategy for CI speech processing [19]. The AEC strategy was proposed from the adaptive wide-dynamic-range compression (AWDRC) amplification scheme used for HA users [20]. It aims to optimize the CR while confining the compressed amplitude of the envelope within a preset DR. To this end, the AEC strategy involves updating the CR in real time by a step size, which determines adaptation rate, in order to increase the (local) DR of output envelope waveform. In other words, through the AEC process, the local DR of speech envelope could be increased, thus yielding a larger modulation depth. Previous studies suggested that the modulation depth is an important factor to speech perception, especially under noisy condition [6, 11, 12]. Therefore, the increased modulation depth may account for the better intelligibility of the AEC-processed speech. Consistent with the success achieved among HA users, the AEC strategy significantly improved the intelligibility of enveloped-based speech (i.e., simulating CI speech processing) when compared to the SEC strategy in both noise [19] and reverberation [21].

In the AEC strategy, the step size is used to control the update rate of CR according to signal fluctuation. Step size is related to the response time of the AEC strategy, and is similar to the attack time (AT) and release time (RT) of wide-dynamic-range compression (WDRC) amplification scheme in HAs [22]. AT and RT describe the durations required for HA device to respond to a changing input signal [23]. A short time constant (i.e., AT or RT) will cause the gain to fluctuate rapidly and generate an undesirable pumping effect; while a large time constant can induce a perception of lagging. Many earlier studies explored the effects of optimal AT/RT values for achieving high intelligibility and satisfactory sound quality in HA users [24–29]. Their results indicated that a fast AT could avoid sudden and transient sounds from becoming too loud, while a slow RT could prevent the distortion of output signals [22]. AT and RT should be optimized based on the performances of speech intelligibility, listening comfort, and sound quality in the amplification process. For the same reason, the step size in the AEC strategy is an important parameter controlling the update rate of CR over short time periods. Therefore, the first aim of the present study is to determine the effect of step size on the intelligibility of AEC-processed speech in noise.

Noise reduction (NR) is an essential part of the modern CI speech processor for improving sound quality/intelligibility in noisy conditions [30–33]. Numerous NR approaches proposed and deployed in CI devices can be broadly divided into multiple- and single-microphone NR approaches. The benefit of multi-microphone NR becomes apparent when the target and noise are spatially separated. The direction of arrival of sound is exploited to spatially filter the signal and successfully remove the noise. van Hoesel and Clark [30] developed an adaptive beam-forming algorithm that used signals from two microphones to attenuate the noise signals; their algorithm had been confirmed to bring substantial benefits to CI users when reverberation was present. Hamacher et al. [32] tested an interchannel processing approach to generate improved monaural output signals and demonstrated that it considerably improved the speech intelligibility of CI users in adverse everyday-life listening conditions. Spriet et al. [34] evaluated the benefit of the two-microphone adaptive beamformer BEAM in the Nucleus Freedom CI system for speech understanding in background noise by CI users. The results confirmed that the adaptive NR approach BEAM in the Nucleus Freedom CI system may significantly increase the speech perception by CI users in noisy conditions. Hersbach et al. [35] tested a combination of NR approach designed to improve CI performance in noise, where based on signal to noise ratio (SNR) estimation was evaluated in combination with several directional microphone approaches available in the Cochlear CP810 sound processor. The results indicated that multi-microphone directionality was effective in improving speech understanding in spatially separated noisy conditions. Later on, Hersbach et al. [36] proposed a beamformer post-filter approach designed to improve beamformer NR performance. Their results suggested that a substantial improvement in performance could be gained for CI users in noisy conditions, where the noise was spatially separated from the target speech. More recently, Buechner et al. [37] investigated the performance of monaural and binaural beamforming technology with an additional NR approach in CI recipients. The study showed that both the adaptive and binaural beamformers were significantly better than the omnidirectional condition for CI users.

Although multi-microphone approaches have shown substantial benefit in previous studies, two limitations have been reported: (1) the performance may degrade in reverberant environments and their usefulness is restricted to acoustic situations where the target and noise are spatially separated [33]; (2) a secondary microphone headphone could be required and thus an increased hardware cost (e.g., two microphones) is necessary. Compared with multi-microphone counterparts, the single-microphone NR approaches are cosmetically more appealing and economically more feasible. Therefore, single-microphone NR approaches are still popularly used in CI processors. Hochberg et al. [38] examined the application of the so-called INTEL method to a single-microphone CI device to perform NR and found that it significantly improved the phoneme recognition threshold of CI receivers in various SNR conditions. Yang and Fu [39] proposed a combination of pause detection and nonlinear spectral subtraction schemes to perform NR for CI patients, and observed significant benefits in sentence recognition in speech-spectrum shaped noise (SSN) conditions at various SNR levels. Loizou et al. [31] assessed the effectiveness of a subspace-based NR approach (Karhunen–Loéve transform, so-called KLT) [40] in CI devices and noted significant sentence recognition improvements in the presence of stationary noise. Hu and Loizou [41] tested a SNR-weighting-based noise-suppression algorithm by directly manipulating the temporal envelopes and found that it produced significant improvements in speech intelligibility in various noisy conditions. Nathalie et al. [42] evaluated the ClearVoice NR approach [43] with young CI recipients; their results demonstrated that children benefited from using ClearVoice in their daily life. Dawson et al. [44] and Hersbach et al. [35] evaluated the SNR-based NR approach on CI recipients; the results indicated that the SNR-based NR approach significantly improved sentence perception for CI recipients in different types of competing noise. More recently, Chen et al. [45] evaluated various single-microphone NR approaches for Mandarin-speaking CI listeners and confirmed that Mandarin speech recognition could also be effectively improved in the presence of various environmental noises.

Chung [46] found that the NR approach greatly enhanced the modulation depth of noise-suppressed signals, but that the benefits (i.e., the enhanced modulation depth) decreased when using more compression. In other words, the overall benefits of an NR approach can be improved by using a suitable compression strategy. These previous studies of NR have found benefits by integrating the NR approach with the SEC and ACE strategies. However it has not been verified whether the AEC strategy can also provide improvements of speech understanding when integrated with an NR approach in noise for CI speech processing. Therefore, the second aim of this study is to evaluate how the envelope compression process interacts with NR approaches in noise. More specifically, we will investigate whether the advantage of NR processing could be preserved when it is integrated with the subsequent AEC strategy, relative to the traditional SEC strategy, and how this advantage would be influenced by factors of input SNR, type of NR, and type of noise.

## Materials and Methods

### Ethics statement

This study was approved by the Human Research Ethics Committee for Non-Clinical Faculties of the University of Hong Kong (EA440114). All participants provided their written informed consent (approved by ethics committees) to participate in this study.

### Signal processing

#### Vocoder-based speech synthesis

Although the number of CI recipients has greatly increased in recent years, one key challenge in the field of CI study is the difficulty of conducting experiments on real CI recipients, especially for some particular regions (e.g., China) where most recipients are children. To handle this issue, vocoder simulations derived from CI speech processing strategies have been presented to normal-hearing (NH) listeners in an attempt to predict the intelligibility pattern of CI speech processing [47]. Many studies have shown that vocoder simulations could predict the pattern of the performance observed in CI users, including the effects of background noise [48], type of speech masker [49], and number of electrodes [3, 50, 51], etc. Note that vocoder simulations are not expected to predict the absolute performance level of each CI user, but rather the trend in performance when a particular parameter is varied, which makes them an extremely valuable tool in the CI field. The present study also conducted speech recognition experiments using vocoder simulations and NH subjects.

**Fig 1:**
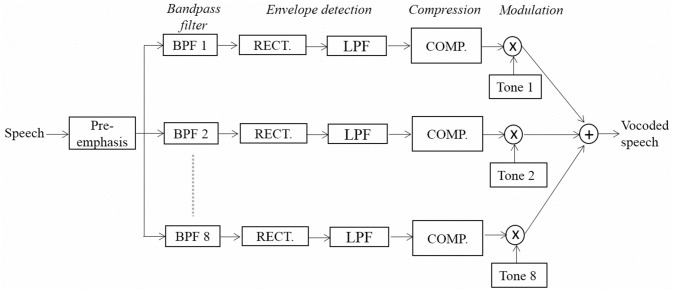
Block diagram of implementing an eight-channel tone-vocoder.

**Fig 2:**
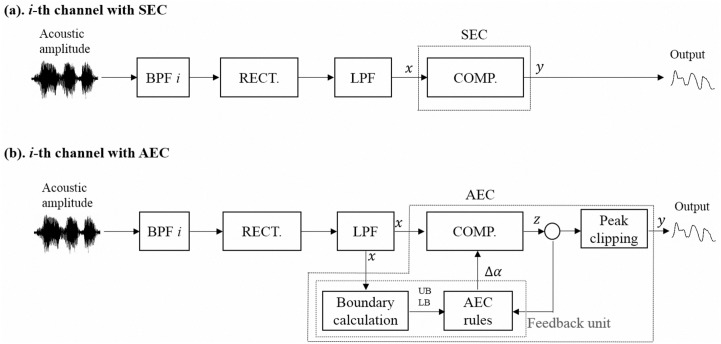
Block diagrams to extract the envelope waveform in the *i*-th channel with the (a) SEC and (b) AEC strategies.

Fig 1 shows the block diagram of an eight-channel tone-vocoder used in this study. The input signal was first processed through the pre-emphasis filter (with a 3 dB/octave roll-off and 2000 Hz cutoff frequency). The bandpass filters (sixth-order Butterworth filters) then filtered the emphasized signal into eight frequency bands between 80 and 6000 Hz (with cutoff frequencies of 80, 221, 426, 724, 1158, 1790, 2710, 4050, and 6000 Hz). The temporal envelope of each spectral channel was extracted by a full-wave rectifier followed by a lowpass filter (second-order Butterworth filter with a 400 Hz cutoff frequency). The envelope of each band was then compressed by the SEC and AEC strategies in this study (see Fig 2 in the next section). The SEC strategy used a fixed CR so as to confine the DR of the amplitude of the entire envelope to within a preset value; while the AEC strategy continuously varied its CR on a frame-by-frame basis (e.g., 2.5 ms in this study), with the maximum and minimum values of the compressed amplitude limited within a preset range. The compressed envelopes then modulated the amplitudes of a set of sine waves (i.e., tone *i* in Fig 1), with frequencies equal to the center frequencies of the bandpass filters. Finally, the amplitude-modulated sine waves of the eight bands were summed, and the level of the summed signal was adjusted to yield a root-mean-square (RMS) value equal to that of the original input signal.

#### Implementation of the SEC strategy

Several methods have been proposed for adjusting the DR of the amplitude of the envelope waveform (also referred to henceforth as “amplitude envelope”). This study adopted the approach developed by Loizou et al. [8] and Chen et al. [12]. In Fig 2(a), *x* and *y* denote the input and output envelope signals, respectively. The output compressed amplitude envelope signal y is computed as:

$$\text{y}=\alpha \times \left(x-\bar{x}\right)+\bar{x,}$$ (1)

where $\bar{x}$ is the mean of the input amplitude envelope *x*, and *α* is a scaling factor (SF) chosen in order to make the output amplitude envelope fall with a certain DR, as:

$$\text{UB}=\text{LB}\times 10^{\frac{\text{DR}}{20}},$$ (2)

where UB and LB are the upper bound (i.e., maximum) and lower bound (i.e., minimum) of the output amplitude values, respectively. It is clear that the mean value of the output amplitude envelope equals the mean value of the input amplitude envelope (i.e.,$\;\bar{y}\;=\;\bar{x}$), regardless of the value of DR selected. Chen et al. [12] found that by empirically setting α to 1/3, 1/5, and 1/13, the DRs of multichannel amplitude envelopes of the Mandarin version of sentences for the Hearing in Noise Test (MHINT) [52] were adjusted to 15, 10, and 5 dB, respectively.

Note that a small SF in Eq (1) denotes a large compression ratio (CR), and vice versa. When *α* equals 0 in Eq (1), the compressed amplitude envelope becomes a DC signal with a constant value of $\bar{x}$ (i.e., $\bar{y}\;=\;\bar{x}$), and the DR is 0 dB; on the other hand, when *α* equals 1 in Eq (1), the output amplitude envelope has the same DR as that of the input amplitude envelope. Fig 2(a) shows a block diagram of the SEC-based speech processor in one channel. Note that a fixed *α* is applied here to the whole amplitude envelope so as to confine its DR to a preset value.

#### Implementation of the AEC strategy

Fig 2(b) shows the block diagram of the AEC-based speech processor in one channel. Comparing Fig 2(a) and 2(b), it is observed that the AEC strategy includes a feedback unit (i.e., to calculate the bounds and apply the AEC rules) and peak clipping unit in each channel. In Fig 2(b), *x*, *z*, and *y* denote the input, compressed, and output envelopes, respectively, and Δ*α* is the step size. Instead of using a fixed SF, the AEC strategy adjusts the SF for each frame (2.5 ms in this study) and computes the compressed amplitude envelope as:

$$z_{t}=\alpha_{t}\times \left(x_{t}-\bar{x}\right)+\bar{x},$$ (3)

where *z*_*t*_ and *x*_*t*_ are the compressed and original envelopes, respectively, at the *t*-th frame, and *α*_*t*_ is the adaptive SF that may be increased or decreased by Δ*α*:

$$\alpha_{t+1}=\alpha_{t}+\Delta \alpha .$$ (4)

Boundary calculation and AEC rules units in Fig 2(b) are used to determine *α*_*t*_ in Eq (3). Given an input envelope, boundary calculation involves first computing the DR of the compressed envelope by estimating two bounds (i.e., upper bound UB and lower bound LB):

$$\{\begin{array}{c} UB=\bar{x}+\alpha_{0}\times \left(max\left(x\right)-\bar{x}\right)\\ LB=\bar{x}+\alpha_{0}\times \left(min\left(x\right)-\bar{x}\right) \end{array},$$ (5)

where max(*x*) and min(*x*) are the maximum and minimum values of input amplitude envelope *x* (i.e., from the entire set of MHINT sentences in this study), and *α*_0_ is an initial SF for AEC. In this study we chose the fixed compression rate used for SEC [*α* in Eq (1)] as the initial compression rate for AEC [*α*_0_ in Eqs (3 and 5)].

With the estimated UB and LB, Δ*α* in Eq (4) is determined by two AEC rules:

1. *Increasing-envelope rule*: The purpose of this rule is to keep the compression processing as close to linear (i.e., *α* = 1) as possible. By doing so, less of the input signal will be perturbed by compression when producing the output signal. When *z*_*t*_ lies between UB and LB, AEC will increase *α*_*t*_ once by using a positive Δ*α* in Eq (4), and thus the SF becomes larger. This increasing-envelope rule stops if *α*_*t+1*_ reaches 1, which corresponds to the original signal being used as the output signal (i.e., no compression being applied).
2. *Decreasing-envelope rule*: The purpose of this rule is to ensure that the amplitude of the output envelope will not be outside the preset DR (i.e., between LB and UB). When *z*_*t*_ is larger than UB or lower than LB, AEC will decrease *α*_*t*_ by a negative Δ*α* increment in Eq (4), and thus the SF becomes smaller. This decreasing-envelope rule stops if *α*_*t+1*_ reaches the initial value (i.e., *α*_0_).

The AEC strategy applies the above two rules to adjust the SF value so as to compress the input acoustic amplitude to fit the DR of the electrical current applied to the CI. However, sudden changes in the input envelope can still cause overshooting or undershooting issues. The peak clipping unit is used, as shown in Fig 2(b), to confine the output envelope to the maximum and minimum levels, where UB and LB are used as the maximum and minimum levels, respectively, in this study. Then, the compressed amplitude envelope is computed as:

$$\{\begin{array}{ll} y_{t}=z_{t}\;, & \;if\;LB<z_{t}<UB\\ y_{t}=UB, & if\;z_{t}\geq UB\\ y_{t}=LB, & f\;z_{t}\leq LB \end{array}.$$ (6)

**Fig 3:**
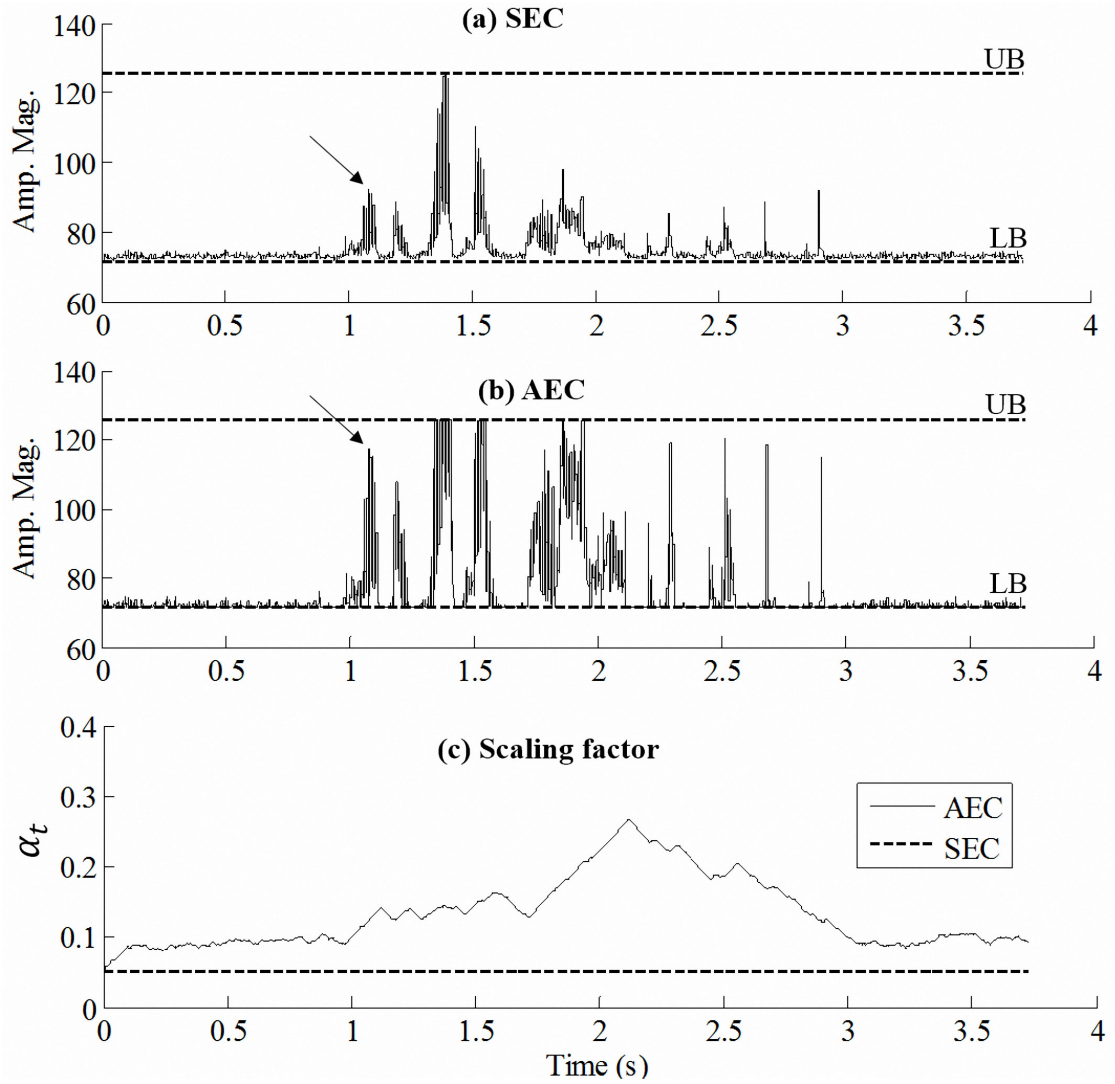
Examples of amplitude envelope processed by the (a) SEC and (b) AEC (i.e., step size was 0.001 per 2.5ms) strategies. The envelope waveform was extracted from the 6th channel of a testing sentence masked by SSN masker at 0 dB, and compressed to a DR of 5 dB. In (c), the solid line shows the SF *α*_*t*_ used in the AEC strategy for the compressed amplitude envelope in (b); the dashed line indicates the fixed SF used in the SEC strategy in (a).

Fig 3(a) and 3(b) show examples processed by the SEC and AEC strategies, respectively, for the amplitude envelope of the 6th channel in the same test condition [i.e., masked by speech-shape noise (SSN) at 0 dB SNR]. The UB and LB in this example are 125.3 and 70.6, respectively, yielding a DR of around 5 dB. Both SEC and AEC strategies aim to compress the DR of the amplitude envelope within this range, as shown in Fig 3(a) and 3(b); however, the SEC strategy applies a fixed SF of α = 1/13, while AEC instead continuously adjusts its SF α_*t*_, as shown in Fig 3(c). Fig 3 indicates that the local (e.g., around 1.1 s) DR is larger for the envelope processed by the AEC strategy (i.e., DR is 4.3 dB) [in Fig 3(b)] than for that processed by the SEC strategy (i.e., DR is 2.3 dB) [in Fig 3(a)]. Fig 3(c) also shows that SF *α*_*t*_ for AEC is universally larger than the fixed SF (i.e., *α* = 1/13) employed in the SEC strategy. These results indicate that AEC can adaptively modify the SF based on the characteristics of the input signal so as to optimally utilize the usable DR. It is also noted that the AEC strategy generates an amplitude envelope with a larger DR and hence a larger modulation depth.

### Experiment 1: Effect of adaptation rate on the intelligibility of AEC-processed speech

The goal of this experiment is to determine the effect of step size in Eq (4) on the intelligibility of AEC-processed speech. Three step sizes were evaluated: (1) 0.001 per 2.5 ms, (2) 0.01 per 2.5 ms, and (3) 0.1 per 2.5 ms, which stand for slow, moderate, and fast update rates and are denoted as 0.001, 0.01 and 0.1 in the following context, respectively.

#### Subjects and materials

Eight NH native-Mandarin listeners participated in the listening experiment, which used MHINT sentences as testing materials [52]. All MHINT sentences were pronounced by a male native-Mandarin speaker, with fundamental frequencies ranging from 75 to 180 Hz, and recorded at a sampling rate of 16 kHz. Two types of maskers [SSN and two equal-level interfering male talkers (2T)] were used to corrupt test sentences at SNR levels of 10 and 5 dB, which were chosen according to a pilot study to avoid ceiling and floor effects.

#### Procedure

The experiment was conducted in a sound-proof room, and stimuli were played to listeners through a set of Sennheiser HD headphones at a comfortable listening level. This study compressed the DR of envelope waveform to 5 dB in tone-vocoder simulations, where the values of α of SEC and *α*_0_ of AEC were set to 1/13 [12]. Four different envelope compression methods were used in this experiment, i.e., SEC, AEC with Δ*α* = 0.001, AEC with Δ*α* = 0.01, and AEC with Δ*α* = 0.1. The last three are henceforth referred to as AEC.001, AEC.01, and AEC.1, respectively. Each subject participated in a total of 16 (= 2 SNR levels × 2 types of maskers × 4 envelope compression methods) test conditions. Each condition contained 10 sentences, and the order of the 16 conditions was randomized across subjects. None of the 10 sentences were repeated across the testing conditions. Subjects were instructed to repeat what they heard, and they were allowed to repeat each stimulus twice. The sentence recognition score was calculated by dividing the number of words identified correctly by the total number of words in each testing condition.

### Experiment 2: Effect of noise reduction on the intelligibility of AEC-processed speech in noise

This experiment was designed to investigate whether the advantage of noise reduction processing could be preserved when integrating it with a subsequent AEC strategy, and how this advantage would be influenced by the factors of input SNR, type of NR, and type of noise.

#### Subjects and materials

Eight new NH native-Mandarin listeners participated in this listening experiment. The same MHINT sentences were used to test the performance, with SSN and 2T maskers used to corrupt test sentences. Since the NR method was incorporated, in addition to 5 and 10 dB SNRs, an additional SNR level of 0 dB was also used as a testing condition.

#### Signal processing with NR and compression strategies

**Fig 4:**
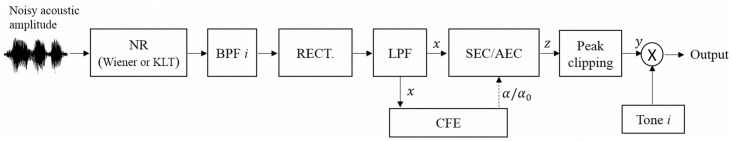
Block diagram to obtain the output tone-vocoded speech for speech enhancement methods followed by a compression strategy (i.e., AEC or SEC) in the *i*-th channel.

An NR stage was inserted before AEC in the eight-channel tone-vocoder. Fig 4 shows the block diagram of the NR and AEC based tone vocoder in one channel. The noisy speech signal was first processed by noise reduction algorithms. This study employed two NR methods, namely Wiener filtering [53] (with moderate noise-suppression capability) and KLT (with strong noise-suppression capability) [40], which represent two most-used types of single-channel NR methods (i.e., the Wiener-filtering approach and the subspace approach). Wiener filter utilizes *a priori* SNR statistics to design a gain function to increase the SNR of speech signals. Due to its simple model structure, Wiener filter provides only moderate NR performance but with the advantage of a relatively low computational cost. For the KLT method, the KLT parts representing the signal subspace were modified by a gain function determined by the estimator, while the remaining KLT parts representing the noise subspace were nulled. The enhanced signal was obtained from the inverse KLT of the modified parts. The techniques used in these two algorithms are detailed elsewhere [40, 53].

Following the NR stage in Fig 4, the envelope of the noise-suppressed signal was extracted by bandpass filtering and waveform rectification. Next, the CR estimation (CRE) stage determined the appropriate SF for SEC or as an initial SF (i.e., *α*_0_) for AEC, based on the input envelope (*x* in Fig 4). The determined SF was then used to transform the output envelope to form a compressed signal (*z* in Fig 4). The final peak clipping stage was used to confine the compressed envelope to be located within the expected DR (*y* in Fig 4). The compressed envelopes then modulated a set of sine waves (i.e., tone *i*) with frequencies equal to the center frequencies of the bandpass filters. Finally, the envelope-modulated sine waves of the eight bands were combined, and the level of the combined signal was adjusted to produce an RMS value equal to that of the original input signal.

**Fig 5:**
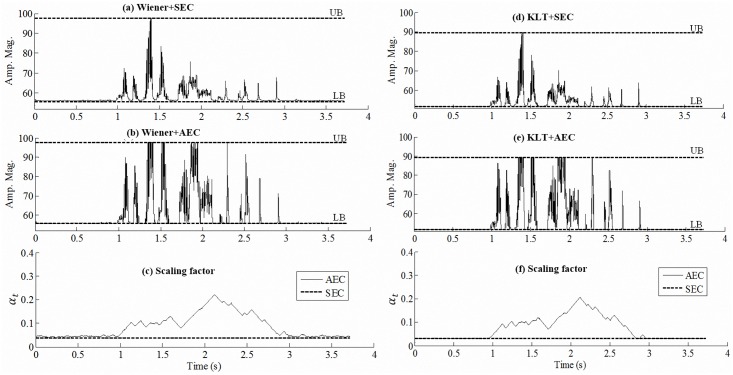
Examples of amplitude envelope for (a) Wiener+SEC, (b) Wiener+AEC, (d) KLT+SEC, and (e) KLT+AEC processing. The envelope was extracted from the 6th channel of a testing sentence masked by SSN masker at SNR 5 dB, and compressed to 5 dB DR. In (c) and (f), the solid lines show the SF used in the AEC strategy [for (b) and (e)]; the dashed lines show the fixed SF in the SEC strategy [for (a) and (d)].

Fig 5 shows examples of the amplitude envelope processed by two NR algorithms (i.e., Wiener filtering and KLT) followed by the corresponding SEC and AEC strategies in the same test conditions. The envelope was extracted from the sixth channel, and compressed to 5 dB DR, where the initial SF was computed by the CRE stage. We note two findings: (1) the KLT performance is similar to that of the Wiener filter integrated with the same compression strategy, and (2) AEC can provide better modulation depth than SEC when it is integrated with the same NR algorithm.

#### Procedure

This experiment followed the same procedure described in Experiment 1. Because NR methods were used in this experiment, the SF should be different from the value used in Experiment 1. As shown in Fig 4, the CRE stage computes α for SEC and α_0_ for AEC based on the NR-processed signals. Four different signal processing methods were used in this set of experiments: (1) Wiener+SEC, (2) Wiener+AEC, (3) KLT+SEC, and (4) KLT+AEC. Please note that the step size (Δα) of AEC was 0.001 in this experiment. Each subject participated in a total of 24 (= 3 SNR levels × 2 types of maskers × 4 types of signal processing) test conditions. Each condition contained 10 sentences, and the order of the 24 conditions was randomized across subjects. None of the 10 sentences were repeated across the testing conditions. Subjects were instructed to repeat what they heard, and they were allowed to repeat each stimulus twice.

## Results

### Experiment 1

**Fig 6:**
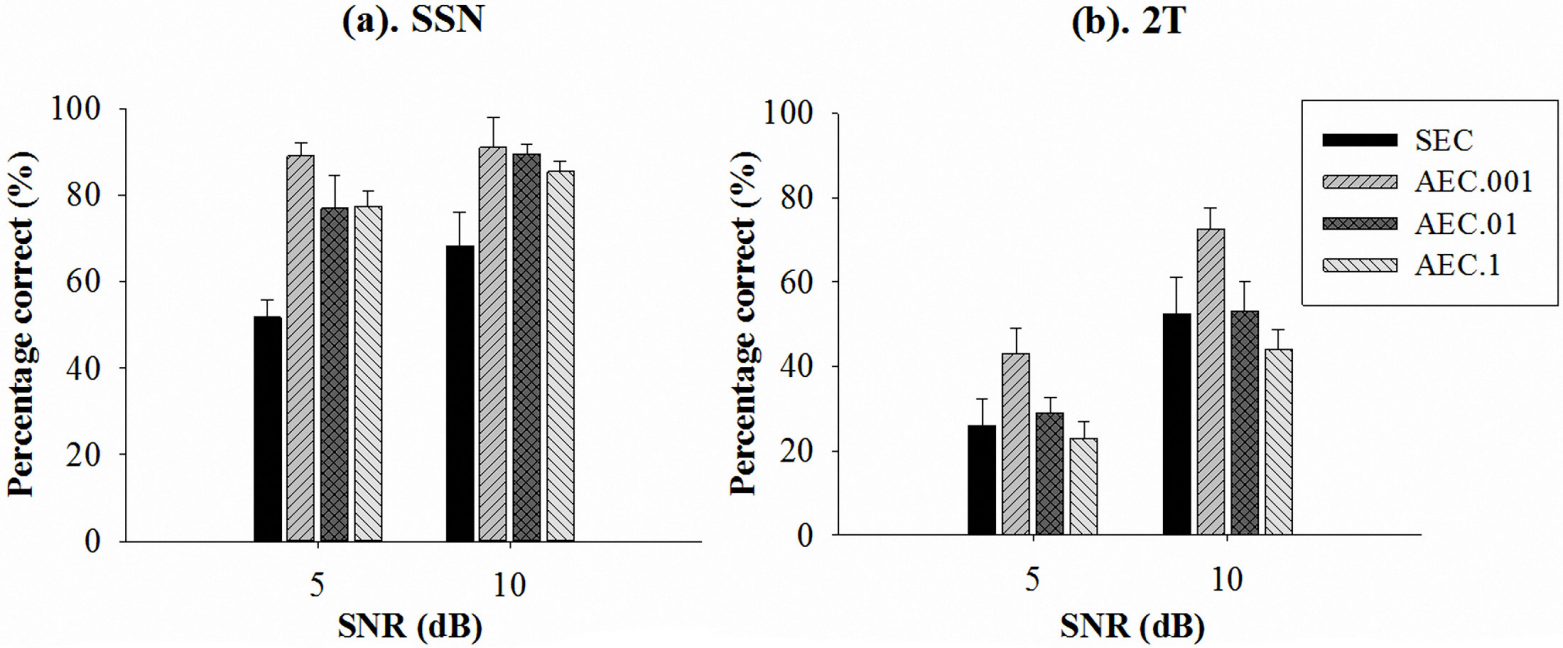
Mean recognition scores for Mandarin sentences with (a) SSN masker and (b) 2T masker at SNR 5 and 10 dB. Each error bar indicates one standard error of the mean (SEM).

Fig 6 shows the speech intelligibility scores (in terms of percentage correct rates) of all test conditions. Fig 6(a) demonstrates that the three AEC strategies (AEC.001, AEC.01, and AEC.1) all give notably higher intelligibility scores than that of the SEC strategy in the SSN condition. Meanwhile, Fig 6(b) shows that the AEC.001 strategy achieves notably higher intelligibility scores than that of the SEC strategy in the 2T masker condition. To further confirm the significance of improvements, the one-way analysis of variance (ANOVA) and Tukey post-hoc comparisons were used to analyze the results of the four compression strategies in the four test conditions. The analysis results are presented in Table 1. In the table, each mean score represensts the corresponding percentage correct score in Fig 6, and *n* denotes the sample size. For the SSN masker, the ANOVA results confirmed that the intelligibility scores differed significantly across the four groups, with (*F* = 10.25, *p* < 0.001) and (*F* = 5.80, *p* = 0.003) at SNRs of 5 and 10 dB, respectively; the Tukey post-hoc comparisons further verified the significant differences for the following group pairs at both SNRs of 5 and 10 dB: (SEC, AEC.001), (SEC, AEC.01), and (SEC, AEC.1). Meanwhile, the ANOVA results for the 2T masker confirmed that the intelligibility scores differed significantly across the four groups, with (*F* = 3.00, *p* = 0.048) and (*F* = 3.49, *p* = 0.029) at SNRs of 5 and 10 dB, respectively; the Tukey post-hoc comparisons verified the significant difference for the following group pair at both SNRs of 5 and 10 dB: (SEC, AEC.001).

**Table 1:** Mean scores of speech intelligibility for different strategies, where each factor was included in the one-way ANOVA and Tukey post-hoc testing.

Test condition	Strategy	*n*	Mean score	*F*	*p*	Post-hoc comparison* (group_*i*_, group_*j*_)
SSN (SNR = 5 dB)				10.25	<0.001	(SEC, AEC.001), (SEC, AEC.01), (SEC, AEC.1)
	SEC	8	51.8			
	AEC.001	8	89.1			
	AEC.01	8	76.9			
	AEC.1	8	77.4			
SSN (SNR = 10 dB)				5.80	0.003	(SEC, AEC.001), (SEC, AEC.01), (SEC, AEC.1)
	SEC	8	68.3			
	AEC.001	8	91.0			
	AEC.01 AEC.1	8 8	89.5 85.4			
2T (SNR = 5 dB)				3.00	0.048	(SEC, AEC.001)
	SEC	8	25.9			
	AEC.001	8	43.2			
	AEC.01 AEC.1	8 8	29.2 23.0			
2T (SNR = 10 dB)				3.49	0.029	(SEC, AEC.001)
	SEC	8	52.5			
	AEC.001	8	72.5			
	AEC.01	8	53.1			
	AEC.1	8	52.5			

* Mean difference is significant at the α = 0.05 level.

### Experiment 2

**Fig 7:**
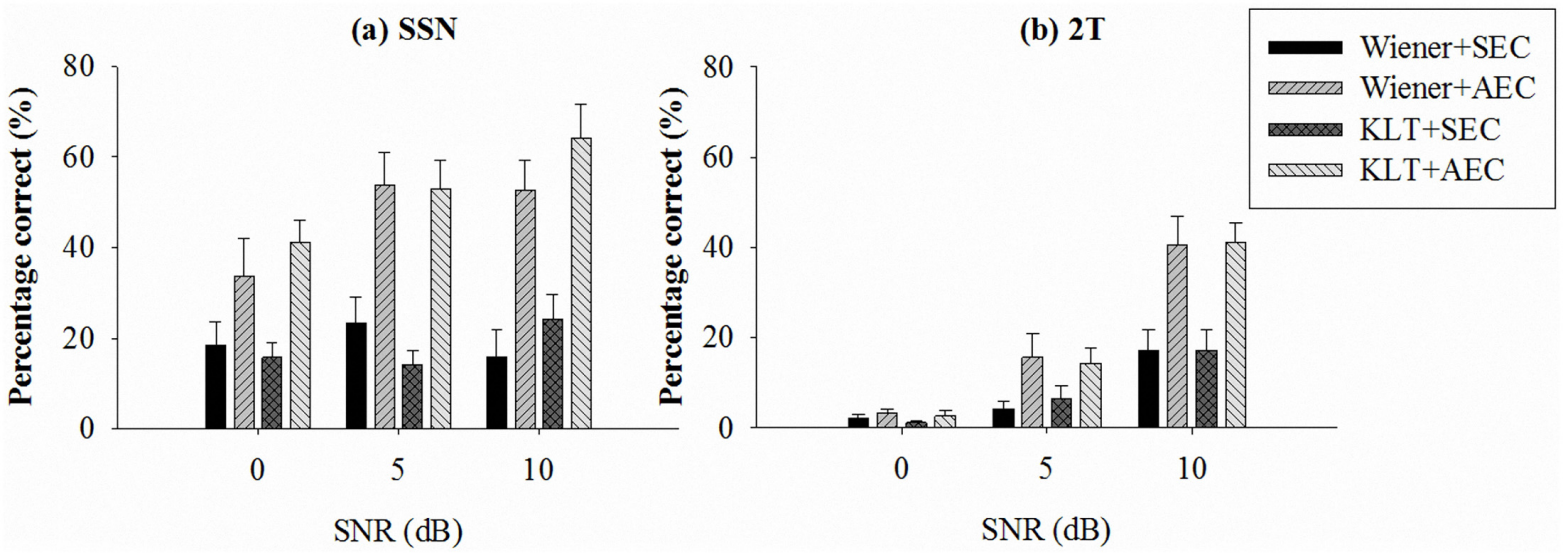
Mean recognition scores for Mandarin sentences with (a) SSN masker and (b) 2T masker, at SNR levels of 0, 5, and 10 dB. The error bars indicate SEM values.

Fig 7 shows the speech intelligibility performance of all test conditions. In the SSN masker conditions, the mean percentage correct rates for conditions of Wiener+SEC, Wiener+AEC, KLT+SEC, and KLT+AEC, respectively, are 18.4%, 33.6%, 15.8%, and 41.1%, for 0 dB SNR, 23.3%, 53.9%, 14.1%, and 53.0% for 5 dB SNR, and 15.7%, 52.6%, 24.1%, and 64.1% for 10 dB SNR. In the 2T masker conditions, the percentage correct rates are 2.3%, 3.1%, 1.1%, and 2.6% for 0 dB SNR; 4.3%, 15.6%, 6.5%, and 14.4% for 5 dB SNR; and 17.1%, 40.6%, 17.3%, and 41.3% for 10 dB SNR. Three-way ANOVA was applied to analyze these results following three factors, i.e., type of masker (masker), SNR level (SNR), and processing method (F1). For model building, the third-order interaction was not significant (*p* > 0.05), so we removed it and ran another model with the three main effects and three second-order interactions. All of them were significant (please refer to Table 2; e.g., F1 was significant with *F*[3,191] = 38.46, *p* < 0.001). Tukey post-hoc analysis showed significant differences for the following group pairs: (Wiener+SEC, Wiener+AEC), (Wiener+SEC, KLT+AEC), (Wiener+AEC, KLT+SEC), and (KLT+SEC, KLT+AEC).

**Table 2:** Mean scores of speech intelligibility for different strategies, where three factors [types of masker (masker), SNR levels (SNR), and processing method (F1)] were included in the three-way ANOVA and Tukey post-hoc testing.

Source of variance	Type III sum of squares	*df*	Mean square	*F*	*p*	Post-hoc comparison (group_*i*_, group_*j*_)
**Corrected model**	65113.9^a^	17	3830.2	20.03	<0.001	(1,2), (1,4), (2,3),(3,4)
**Intercept**	110544.0	1	110544.0	578.15	<0.001	
**SNR × F1**	3800.57	6	633.43	3.31	0.004	
**masker × F1**	4892.81	3	1630.94	8.53	<0.001	
**masker × SNR**	2508.07	2	1254.04	6.56	0.002	
**masker**	19784.38	1	19784.38	103.47	<0.001	
**SNR**	12065.76	2	6032.88	31.55	<0.001	
**F1**	22062.35	3	7354.12	38.46	<0.001	
**Error**	33269.05	174	191.20			
**Total**	208927.00	192				
**Corrected total**	98382.99	191				

F1 group variable: 1, Wiener+SEC; 2, Wiener+AEC; 3, KLT+SEC; 4, KLT+AEC.

**Dependent variable:** speech intelligibility scores.

^a^ *R* ^2^ = 0.669 (adjusted *R* ^2^ = 0.624)

## Discussion and Conclusions

The present study has two aims: (1) to investigate the effect of step size on the intelligibility of AEC-processed speech; and (2) to determine the compatibility of AEC with the NR algorithms under various noisy conditions.

For the first aim, we have presented Fig 3 to qualitatively compare the signal processing effects of the AEC and SEC strategies and reported the results of Experiment 1 to quantitatively analyze the correlation of step size and the intelligibility of AEC-processed speech. Fig 3(c) indicates that (1) the AEC strategy varies the SF by Δ*α* based on the characteristics of input signals so as to optimally utilize the usable DR, and (2) the SF for AEC is larger than the initial value (*α*, the static SF used in the SEC strategy). Using a large SF *α*_*t*_ in Eq (3) could provide a larger DR for the envelope of the electrical signal supplied to the CI. Larger modulation depths for AEC than for SEC can be observed by comparing Fig 3(a) and 3(b) in the same noisy condition. Previous studies have suggested that modulation depth is an important factor for speech perception, especially in noisy conditions [47, 54]. The present results therefore imply that the AEC strategy can provide better speech intelligibility than SEC.

**Fig 8:**
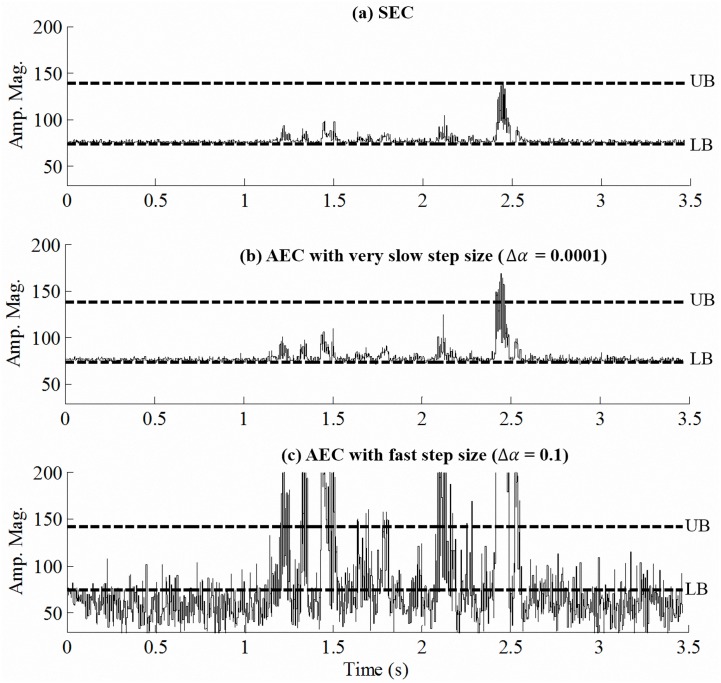
Examples of amplitude envelope processed by (a) SEC, (b) AEC with a very slow step size (i.e., Δ*α* = 0.0001), and (c) AEC with a fast step size (i.e., Δ*α* = 0.1). The envelope was extracted from the 6th channel of a testing sentence masked by SSN at 5 dB, and compressed to 5 dB DR.

The results of Experiment 1 further indicate that the value of adaptation rate Δ*α* affects the intelligibility of AEC-processed speech. As noted, the SF is an important parameter in the AEC strategy. The use of an inappropriate value of Δ*α* may reduce the speech intelligibility benefits, especially in test conditions with interfering masker (i.e., 2T masker in this study). When a very small value of Δ*α* is used in the AEC strategy, it is difficult to obtain a larger SF for short-term signals, thereby also making it difficult to obtain a larger modulation depth from AEC. On the other hand, the use of a very large value of Δ*α* may induce the perception of pumping and increase the probability of the processed envelope falling within the range of peak clipping and thereby causing distortion. Therefore, the value of adaptation rate needs to be selected as a trade-off between speech intelligibility and the pumping effect and distortion. Fig 8 exemplifies results obtained by traditional SEC, AEC with a very slow step size (i.e., Δ*α* = 0.0001), and AEC with a fast step size (i.e., Δ*α* = 0.1). This example shows that using a very small value of Δ*α* [0.0001 in Fig 8(b)] limits the benefit of AEC processing, because the step size is too small over a short time period to provide a sufficiently large modulation depth relative to the traditional SEC strategy. In contrast, when the value of Δ*α* is set too large [0.1 in Fig 8(c)], the step size is too large over a short time period, which will result in a considerable amount of envelope information appearing outside of the DR and thus cause distortion by peak clipping and decrease the intelligibility of AEC-processed speech. In addition, when the envelope waveform varies too rapidly in the AEC strategy, it will also cause the perception of pumping to affect the performance of speech intelligibility. The results of Experiment 1 indicate that, on average, Δ*α* = 0.001 provides a higher speech intelligibility score than the other values (i.e., Δ*α* = 0.01 and 0.1), especially in test conditions with 2T masker. Therefore, Δ*α* = 0.001 strikes the optimal balance among benefits, pumping effect, and distortion in the AEC strategy. Since we only used these three values of Δ*α* (i.e., slow, moderate, and fast step size) to investigate the performance of the AEC strategy in this study, future studies should further investigate its effect while considering the wider characteristics of language (e.g., the band importance function or tone of Mandarin) and noise types. In addition, the technology of machine learning could be used to help tune this parameter in future studies.

Results showed that the AEC strategy can adaptively adjust SF to fully use the DR of CI recipients. As presented earlier, the ACE sound coding strategy processes speech signals by using front-end AGC and ADRO to adjust the gain and section of speech signals in each frequency band; the processed signals are then compressed to fit the available range of stimulation current by LGF (similar to the SEC strategy), where a fixed SF is used. On the other hand, AEC dynamically reduces CR [by increasing SF *α*_*t*_ in Eq (3)] when DR is sufficient, and increases CR [by reducing SF *α*_*t*_ in Eq (3)] when DR is insufficient. It can be noted that the proposed AEC and ACE (front-end AGC, ADRO, and LGF) operate differently: When the DR of the CI user is larger than the (local) DR of input signal, ACE will adjust the gain and section of speech signals while AEC will directly adjust SF to maximize the DR. In real-world CI systems, the UB and LB will be set based on each user’s comfortable current (C-level) and threshold current (T-level), and thus AEC does not need to prepare the intensity statistical distribution (which is required in ADRO). In the meanwhile, we believe that AEC can be suitably integrated with AGC (to avoid the clipping effects instead setting UB and LB in Eq (6)) and ADRO (to select proper sections of the speech signals) to provide even better speech intelligibility. The integration of AEC and existing successful approaches will be studied in the future.

For the second aim, we have demonstrated Fig 5 to qualitatively compare the signal processing effects of the AEC and SEC strategies integrated with NR approaches and reported the results of Experiment 2 to quantitatively investigate the compatibility of AEC with NR algorithms under various noise conditions. From Fig 5, we noted the same observations as those from Fig 3: AEC can provide wider modulation depth than SEC when integrated with the same NR method, implying that the AEC strategy can provide better speech intelligibility than SEC under noisy conditions. The results of three-way ANOVA and Tukey post-hoc comparisons performed on the data collected in Experiment 2 indicated that AEC could produce higher speech intelligibility scores than SEC when integrated with both Wiener filter and KLT. The reasons for the SEC strategy not performing as well as AEC in noisy conditions may be similar to those for hearing aids [55, 56], that is the static compression processing will increase the low-level noise during the pauses in the speech signal and thereby decrease the SNR performance for the NR algorithm. Hence, SEC maintains relatively less benefits from the NR algorithm when compared with the AEC strategy. In addition, the results of Experiment 2 (i.e., Table 2) show that different NR algorithms (i.e., Wiener filter or KLT) did not produce significant differences when integrated with the same compression strategy (i.e., SEC or AEC). In contrast, different compression strategies produced clearly different results when integrated with the same NR algorithm, where AEC outperformed SEC consistently. The main reason for NR algorithms not producing significant differences is probably the very narrow electrical DR of CIs (i.e., 5 dB). Processing noisy signals by an NR algorithm will increase the input DR of speech. This requires the use of a smaller SF to ensure that the sound signals remain within the audibility range (i.e., between UB and LB). From the examples of Figs 3(c) and 6(c), the initial SF (i.e., *α*_0_) was larger without NR than with NR in the same test conditions, and the speech intelligibility scores were higher for a larger SF. This provides further evidence that the compression strategy is more important than using the NR algorithm in speech-in-noise conditions.

In conclusion, this study investigated the performance of the AEC strategy with the aims of observing how its performance is affected by the step size and how well it works with NR algorithms. From the experimental results, it can be concluded that (1) adaptation rate is an important parameter in the AEC strategy, and a suitable step size (i.e., Δ*α* = 0.001) strikes a good balance of speech intelligibility, pumping effect, and distortion in the AEC strategy; (2) the AEC strategy provides better speech intelligibility than the traditional SEC strategy when integrated with NR algorithms; and (3) the AEC strategy is suitable for integrating with NR methods. In summary, this study confirms that the AEC strategy is a highly promising approach to enhance the understanding of speech in the presence of noise for CI speech processing. A limitation of this study was that the listening experiments involved tone-vocoded speech simulations only and the AEC performance was not tested in actual CI devices. Although the simulations using vocoders can be powerful in that some effects of CI processing can be evaluated in the healthy auditory system of NH listeners while bypassing subject-dependent factors associated with hearing loss and cochlear implantation, and have many success in predicting outcome (e.g., [3] [48–51, 57]), they still have potential risks when transferred to the real CI listeners. For example, a particular CI user may only have a 5 dB DR between their T-level and C-level. However it should be noted that C-level is very loud for the CI recipient, and T-level is very soft; hence, the difference in loudness is very much larger than an NH listener would perceive for a sound pressure level difference of 5 dB. In other words, compressing acoustic levels into a 5 dB range provides NH listeners with much smaller loudness differences than a CI recipient experiences. Therefore, future work will be evaluating the investigated methods based on speech intelligibility tests with real CI subjects in proper clinical situations.
